# The Opium Problem

**Published:** 1894-02-24

**Authors:** 


### THE OPIUM PROBLEM.

Dr. Harford Batterby writes: There are two sides to this as to every other question, and as you have clearly put one side, perhaps you will allow me to put before your readers some of the reasons which have led some of us to take the other side, and that, as we think, on the truest scientific grounds.

You have spoken exclusively of India, but it is not there that the chief evil exists, but in China, to which country by far the greater proportion of British opium is sent. We have incontrovertible evidence with regard to opium-smoking in China that it is a vice of the most demoralising character, and one which is ruining morally and physically a very large part of the Chinese nation. Only a few days ago a friend of mine who has just returned from Inland China, a graduate of Cambridge, and a contemporary of my own, said that in one small town in China he had known as many as three deaths from opium-poisoning in one day. Another friend, a graduate of Dublin University, wrote a short time ago to say that the horrors said to be due to opium, far from being exaggerated, could not be adequately stated upon paper; they must be seen to be realised. These are only two from a number of other statements, but I quote these as from men upon whose word I know that I can rely, and since they come from entirely different parts of China. It is well known that one of the first works which has to occupy every missionary to Inland China is the treating of the opium habit, and so opium refuges have been formed in all parts of China. Much then as we deplore the evil of alcohol in our own country, we cannot compare them with the influence of opium in China.

But attention has been drawn from China during the last few months by the establishment of the Opium Commission to India, and it is important to note that this Commission was not asked for by any of the bodies who protest against the opium traffic.

It is well known that opium is not used in India to anything like the same extent as in China (the actual proportion during the last ten years has been 15½ lbs. of opium exported to China for every pound consumed in India), where it is used chiefly for smoking, the most pernicious form of indulgence in opium, whilst in India opium eating is the most common mode of using the drug.

The opinions of European medical practitioners resident in India are of the greatest importance, but they are by no means unanimous upon the main points of this controversy, and evidence given before the Opium Commission cannot always be taken as scientifically accurate; as a matter of fact a very large number of the answers given by medical men were based not upon carefully collected personal observation, but upon vague impressions. It will therefore be well if we refrain from passing too decided an opinion upon the evidence before us until we have the full reports of the proceedings of the Commission.

In the meantime it must be remembered that the main question is a Chinese one. This is not the place to show how dishonourable have been our dealings with China with regard to the importation of opium, and it is no excuse for the continuance of this policy that the Chinese are cultivating opium for themselves. They are doing this, and the revenue from the trade with China is proportionately decreasing. It would be more honourable for England to withdraw from that trade rather than allow it to be taken from her by Chinese competition.

